# The Structure of *Treponema pallidum* Tp0624 Reveals a Modular Assembly of Divergently Functionalized and Previously Uncharacterized Domains

**DOI:** 10.1371/journal.pone.0166274

**Published:** 2016-11-10

**Authors:** Michelle L. Parker, Simon Houston, Charmaine Wetherell, Caroline E. Cameron, Martin J. Boulanger

**Affiliations:** Department of Biochemistry & Microbiology, University of Victoria, Victoria, British Columbia, Canada; University of Kentucky College of Medicine, UNITED STATES

## Abstract

*Treponema pallidum* subspecies *pallidum* is the causative agent of syphilis, a chronic, multistage, systemic infection that remains a major global health concern. The molecular mechanisms underlying *T*. *pallidum* pathogenesis are incompletely understood, partially due to the phylogenetic divergence of *T*. *pallidum*. One aspect of *T*. *pallidum* that differentiates it from conventional Gram-negative bacteria, and is believed to play an important role in pathogenesis, is its unusual cell envelope ultrastructure; in particular, the *T*. *pallidum* peptidoglycan layer is chemically distinct, thinner and more distal to the outer membrane. Established functional roles for peptidoglycan include contributing to the structural integrity of the cell envelope and stabilization of the flagellar motor complex, which are typically mediated by the OmpA domain-containing family of proteins. To gain insight into the molecular mechanisms that govern peptidoglycan binding and cell envelope biogenesis in *T*. *pallidum* we report here the structural characterization of the putative OmpA-like domain-containing protein, Tp0624. Analysis of the 1.70 Å resolution Tp0624 crystal structure reveals a multi-modular architecture comprised of three distinct domains including a C-terminal divergent OmpA-like domain, which we show is unable to bind the conventional peptidoglycan component diaminopimelic acid, and a previously uncharacterized tandem domain unit. Intriguingly, bioinformatic analysis indicates that the three domains together are found in all orthologs from pathogenic treponemes, but are not observed together in genera outside *Treponema*. These findings provide the first structural insight into a multi-modular treponemal protein containing an OmpA-like domain and its potential role in peptidoglycan coordination and stabilization of the *T*. *pallidum* cell envelope.

## Introduction

*Treponema pallidum* subspecies *pallidum* (hereafter *T*. *pallidum*) is a medically important spirochete bacterium and the causative agent of syphilis, a chronic, multistage systemic disease transmitted sexually, through direct contact with an infectious lesion, or *in utero*. It is estimated that the current global burden is 36 million cases with 11 million new infections each year [1]. Congenital syphilis is also a major global health concern with approximately 1.4 million pregnant women infected annually [2]. Furthermore, symptomatic syphilis infections increase the risk of HIV transmission and acquisition 2- to 5-fold [3].

Eighteen years have passed since the publication of the first complete *T*. *pallidum* genome sequence [4], yet the molecular basis of *T*. *pallidum* pathogenesis remains enigmatic. Research efforts are complicated by the fact that *T*. *pallidum* is an obligate human pathogen that cannot be cultured *in vitro* or genetically modified. Moreover, compared to conventional Gram-negative bacteria, *T*. *pallidum* has an inherently fragile cell envelope [5, 6] composed of a chemically distinct peptidoglycan layer that is thinner and more spatially separated from the outer membrane, as well as periplasmic flagella (endoflagella) that are located between the outer membrane and the peptidoglycan layer [5, 6]. These ultrastructural differences suggest that treponemes such as *T*. *pallidum* may harbor divergent molecular strategies for linking the peptidoglycan layer with the inner and outer membranes to stabilize the bacterial cell envelope.

In Gram-negative bacteria, cell envelope stabilization is partially achieved through Outer Membrane Protein A (OmpA)-like domain-containing proteins that bind non-covalently to peptidoglycan. This domain is commonly found in bacterial proteins that support outer membrane integrity and indirectly facilitate host-pathogen interactions by acting as a structural bridge between the peptidoglycan layer and the outer membrane [7–10]. In conventional Gram-negative bacteria the stabilization of the outer membrane via OmpA domain-containing proteins occurs via one of three mechanisms: insertion of an N-terminal beta-barrel domain [11] or an N-terminal lipid anchor distal to the C-terminal OmpA-domain into the outer membrane [12], or association of the OmpA-domain with bacterial outer membrane porins [7–10]. OmpA-like domains that contain peptidoglycan binding sites are also found in bacterial flagellar motor complex proteins, including motility protein B (MotB) [13–15]. MotB is an essential component of the torque-generating stator ring in the flagellar motor complex [16–18] where its N-terminus is anchored in the cytoplasmic membrane via a membrane-spanning helix and its C-terminal OmpA-like domain directed into the periplasm where it coordinates the peptidoglycan layer [13, 15]. It appears that the peptidoglycan-binding OmpA-like domain was acquired by outer membrane-associated proteins and inner membrane-associated flagellar motor complex proteins from a common ancestor long before their protein functions diverged [19]. Accordingly, the OmpA-like domains of these otherwise functionally divergent proteins exhibit significant sequence similarity and share a common structural fold [13, 15, 19].

The *T*. *pallidum* protein Tp0624 is annotated as belonging to the OmpA-OmpF porin (OOP) family [20]. The C-terminal region of Tp0624 is predicted to adopt an OmpA domain architecture, but it is unclear what role a canonical peptidoglycan binding domain might possess in an organism that contains an unusual form of peptidoglycan and has a divergent cell envelope ultrastructure. Further complicating the functional prediction of Tp0624 is the observation that the N-terminal two thirds of the protein does not show significant sequence homology to any functionally- or structurally-characterized protein. The lack of sequence identity is consistent with *T*. *pallidum* being a phylogenetically distinct bacterium with no known orthologs nor assigned functions for almost 30% of its predicted protein-coding genes [4]. Moreover, the extreme genomic reduction in obligate pathogens such as *T*. *pallidum* often translates to the expression of multi-modular proteins with increased functional complexity [21, 22].

*Treponema pallidum* transcriptomic [23] and proteomic [24] studies have shown that Tp0624 is expressed during infection, yet its precise biological role is unknown. The presence of an OmpA-like domain suggests a potential role for Tp0624 in peptidoglycan coordination and cell envelope stabilization, which could be facilitated by the uncharacterized N-terminal region. To gain insight into the function of Tp0624, we first determined its three-dimensional structure, which revealed a trimodular architecture incorporating a highly divergent OmpA-like domain and a previously uncharacterized tandem domain with intriguing implications for peptidoglycan coordination. Collectively, these data provide insight into how the unusual cell envelope structure may be stabilized in the human pathogen *T*. *pallidum*.

## Materials and Methods

### Ethics statement

All animal studies were approved by the local institutional review board at the University of Victoria and were conducted in strict accordance with standard accepted principles as set forth by the Canadian Council on Animal Care (CCAC) and National Institutes of Health in a facility accredited by the American Association for the Accreditation of Laboratory Animal Care.

### Bacterial growth and genomic DNA extraction

*T*. *pallidum* subsp. *pallidum* (Nichols strain) was propagated in, and extracted from, New Zealand White rabbits as described elsewhere [25]. Genomic DNA was purified from treponemes using the DNeasy Tissue kit (Qiagen). All DNA purifications were conducted according to the manufacturer’s instructions for Gram-negative bacteria.

### Cloning, protein production and purification

Eight signal sequence prediction servers were used to predict the presence and position of a potential cleavable signal peptidase type I (SPI) signal sequence within the N-terminus of Tp0624: PrediSi (http://www.predisi.de/index.html; cleavage at T61), Signal BLAST (http://sigpep.services.came.sbg.ac.at/signalblast.html; cleavage at G58), Signal-CF (http://www.csbio.sjtu.edu.cn/bioinf/Signal-CF/; cleavage at A59), Spoctopus (http://octopus.cbr.su.se/; cleavage at G58), SPEPLip (http://gpcr.biocomp.unibo.it/cgi/predictors/spep/pred_spepcgi.cgi; cleavage at A59), Signal3L (http://www.csbio.sjtu.edu.cn/bioinf/Signal-3L/; cleavage at A30), EMBOSS sigcleave (http://www.bioinformatics.nl/cgi-bin/emboss/sigcleave; cleavage at A32), and SignalP (http://www.cbs.dtu.dk/services/SignalP/; SPI signal sequence not fully predicted, but graphical analysis shows a below cut-off cleavage prediction at A59). For expression of the predicted mature full-length protein, a DNA fragment encoding residues T61-D476 of Tp0624 was PCR-amplified from *T*. *pallidum* (Nichols Strain) genomic DNA using the following primers: 5´-CAG ACT GCT AGC ACC CAG GAT GCC GTA CAC ACC G (sense primer with NheI restriction site [underlined]) and 5´-CTG TCT G GC GGC CGC ATC GCG CAA GAT GGT GAT CTC C (antisense primer with NotI restriction site [underlined]). The PCR amplicon was then cloned into a modified pET28a (VWR International) vector with a TEV protease cleavable N-terminal hexa-histidine tag. The construct was confirmed by DNA sequencing. The construct was transformed into the *E*. *coli* expression strain BL21 Star (DE3) (Invitrogen). Transformants were cultured at 37°C in LB broth containing 100 μg/mL ampicillin until the A_600_ reached 0.4. Cultures were induced with 0.4 mM IPTG for 16 h at 25°C and harvested by centrifugation at 4°C. Cell pellets were resuspended in buffer (20 mM HEPES pH 7.5, 500 mM NaCl, 20 mM imidazole) and flash frozen in liquid nitrogen and stored at -80°C.

Purification of recombinant protein was performed using immobilized nickel affinity and size exclusion chromatography (SEC). Briefly, recombinant His-tagged protein was loaded onto 1 ml HisTrap FF affinity columns (GE Healthcare) pre-packed with pre-charged Nickel Sepharose 6 Fast Flow resin. Columns were then washed with buffer (20 mM HEPES pH 7.5, 500 mM NaCl, 20 mM imidazole) and bound recombinant protein was eluted in a buffer consisting of 20 mM HEPES pH 7.5, 500 mM NaCl, and 500 mM imidazole. Tp0624 was then further purified by SEC on a HiLoad^™^ 16/60 Superdex^™^ 75 column (GE Healthcare) pre-equilibrated in HEPES-buffered saline (HBS: 20 mM HEPES pH 7.5, 150 mM NaCl). Notably, Tp0624 eluted from the SEC as a monomer (S1 Fig). The N-terminal His tag was removed by cleavage with TEV protease at 16°C for 16 h. Cleaved protein was then purified by SEC in HBS as described above, and concentrated to 10 mg/mL.

### Crystallization and data collection

Crystals of Tp0624 were originally identified in the MCSG-1 screen (Microlytic) using sitting drops at 18°C. The final, refined drops were set in hanging drops and consisted of 3.6 μL Tp0624 at 10 mg/mL in HBS with 1.8 μL of reservoir solution (0.1 M HEPES pH 6.8, 0.25 M MgCl_2_, 14% PEG3350) and were equilibrated against 300 μL of reservoir solution. Crystals appeared within 24 h and grew to a final size within 3 days. Crystals were cryoprotected in reservoir solution supplemented with 12.5% glycerol and flash cooled in liquid nitrogen. Diffraction data were collected on beamline 08ID-1 at the Canadian Light Source (CLS). For phase determination, Tp0624 was crystallized using the same technique as for the native crystals but the reservoir solution consisted of 0.1 M HEPES pH 6.8, 0.45 M MgBr_2_, 14% PEG3350. Diffraction data were collected on beamline 08ID-1 at CLS using an optimized wavelength of 0.9201 Å for the *f*” bromine edge.

### Data processing, structure solution and refinement

Diffraction data were processed to 1.70 Å (Tp0624) or 1.92 Å (Tp0624-Br) resolution using Imosflm [26] and Aimless [27]. The structure of Tp0624 was solved by Bromide single wavelength anomalous dispersion. A total of 53 high confidence Br sites were identified using ShelxC/D [28] and refined using Phenix.phaser-ep [29]. High quality phases enabled building and registering of approximately 75% of the backbone using Phenix.autobuild [30]. The Tp0624 native structure was solved by molecular replacement using a single Tp0624 chain from the Br-phased model in Phaser [31]. COOT [32] was used for model building and selection of solvent atoms, and the model was refined in Phenix.refine [33]. Complete structural validation was performed with Molprobity [34], including analysis of the Ramachandran plots, which showed greater than 98% of residues in the most favored conformations. Five percent of reflections were set aside for R_free_ calculation. Data collection and refinement statistics are presented in Table 1. The atomic coordinates and structure factors have been deposited in the Protein Data Bank under accession code **5JIR**.

**Table 1:** Data collection and refinement statistics.

	Tp0624-Br SAD	Tp0624 native
Data collection statistics		
Spacegroup	P2_1_2_1_2_1_	P2_1_2_1_2_1_
a, b, c (Å)	62.78, 104.3, 141.3	63.02, 103.50, 140.68
α, β, γ (deg.)	90, 90, 90	90, 90, 90
Wavelength	0.9201	0.9794
Resolution range (Å)	47.11–1.95 (2.00–1.95)	63.02–1.70 (1.73–1.70)
Measured reflections	879019 (58876)	592930 (29342)
Unique reflections	68488 (4574)	101666 (4969)
Redundancy	12.8 (12.9)	5.8 (5.9)
Completeness (%)	100.0 (100.0)	99.9 (100.0)
*I/σ(I)*	12.4 (4.0)	9.4 (2.2)
R_merge_	0.172 (0.745)	0.089 (0.684)
Refinement statistics		
Resolution (Å)		51.77–1.70
R_work_ / R_free_		0.189/0.215
No. of atoms		
Protein (A/B)		3233/3189
Glycerol/Chloride		24/8
Solvent		346
B-values (Å^2^)		
Protein (A/B)		28.1/28.2
Glycerol/Chloride		33.5/24.6
Solvent		29.1
r.m.s. deviation from ideality		
Bond lengths (Å)		0.008
Bond angles (deg.)		1.10
Ramachandran statistics (%)		
Most favoured		98.4
Allowed		1.6
Disallowed		0

Values in parentheses are for the highest resolution shell.

5% of reflections were set aside for calculation of R_free_.

### Bioinformatic identification of Tp0624 orthologs

Orthologs of Tp0624 were identified using BLASTp (PSI-BLAST algorithm), the *T*. *pallidum* query sequence (Tp0624; NCBI accession number WP_010882070) and the NCBI non-redundant database. Tp0624 residues A64-F177, Y198-C317, and D332–D476 were used for the identification of orthologous proteins containing domains with significant homology to Tp0624 domains 1, 2, and 3, respectively. All identified orthologs had an e-value cutoff of 0.00026.

## Results and Discussion

### A non-canonical signal peptide suggests that Tp0624 is exported from the cytoplasm

To assess the potential for Tp0624 to adopt a subcellular localization consistent with canonical OmpA-containing proteins (i.e. exported from the cytoplasm), we first sought to establish the presence of a signal peptide. Of the eight signal sequence servers used, seven predicted a cleavable signal peptidase type I (SPI) signal sequence within the N-terminus of Tp0624. The majority of these programs predict cleavage to occur near residue 60 indicating an unusually long signal peptide. Notably, however, atypically long signal sequences that exhibit diversity in the length of the extreme N-terminal region of the signal peptide have been reported [35]. The assignment of a signal sequence was further supported based on the analysis of the region immediately prior to the predicted cleavage site, which contains the hallmarks of SPI-cleaved signal peptides including a positively charged N-terminal region, a central hydrophobic region, and a neutral/polar C-terminal region [36]. Moreover, an alignment of eighteen Tp0624 homologs (S2 Fig) revealed that significant sequence homology was observed only after the predicted signal peptide indicating that the N-terminus is structurally independent of the protein core. Based on these observations, we revisited our recent proteomic analysis of *T*. *pallidum* isolated from an infected rabbit. Somewhat unexpectedly, we identified a peptide that mapped to the predicted signal sequence region of Tp0624 [24]. While this peptide may have originated from the pre-protein before signal peptidase cleavage, it also may reflect that the signal peptide is not cleaved following export from the cytoplasm resulting in Tp0624 being anchored in the inner membrane via an N-terminal hydrophobic transmembrane segment with its head group directed into the periplasm. Consistent with this possibility is the fact that the predicted signal peptide central region (F18-F52) is very hydrophobic (GRAVY [grand average of hydropathy] value = 1.166) compared to the mature protein (GRAVY value = -0.220). While we are unable to definitively assign a subcellular location for Tp0624, there is strong bioinformatic evidence that Tp0624, like other OmpA-containing proteins, is exported from the cytoplasm.

### Tp0624 adopts an ordered alpha/beta structure

**Fig 1:**
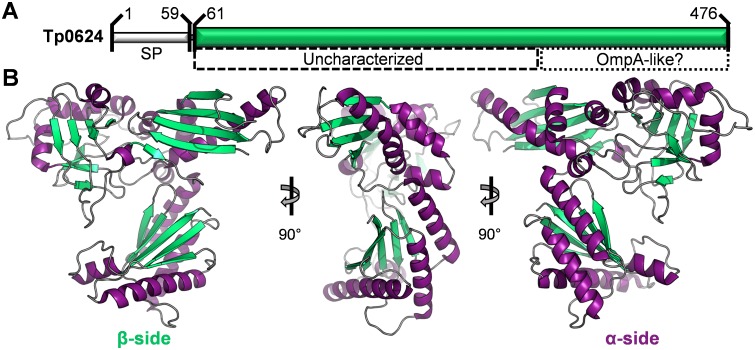
Overall structure of Tp0624. **(A)** Sequence features of Tp0624. SP—signal peptide (approximately residues 1 to 60). Green bar—construct used for structural studies (T61-D476). Dashed box—region with predicted secondary structure elements but no sequence similarity to any characterized protein. Dotted box—putative OmpA-like C-terminal domain. **(B)** Orthogonal views of Tp0624 tertiary structure shown as a cartoon with beta-strands in green, alpha helices in purple, and connecting coil in grey. Note the slightly flattened, triangle-like architecture with two distinct sides defined by either beta-strands (left) or alpha-helices (right).

To establish the optimal start site for the recombinant Tp0624, we integrated secondary structure predictions with the sequence alignment of the eighteen Tp0624 homologs (S2 Fig). Based on these analyses, a construct incorporating T61 to D476 was cloned for expression in *E*. *coli* (Fig 1A). The recombinant hexa-histidine fusion protein was purified by nickel affinity and size exclusion chromatography (SEC), which showed an elution profile consistent with a Tp0624 monomer (S1 Fig). Crystallization trials were set with purified Tp0624 and diffraction data on optimized crystals were collected to a resolution of 1.70 Å. In the absence of a suitable molecular replacement model, crystals of Tp0624 were soaked in bromide, data collected at the optimized wavelength for the *f*” bromine edge, and the structure phased by single wavelength anomalous dispersion (Table 1). The structure revealed that Tp0624 crystallized with two molecules in the asymmetric unit of the primitive orthorhombic unit cell with each monomer related by a root mean square deviation (rmsd) of 0.70 Å over 393 Cα atoms. A minimal interface between the two protomers is consistent with the SEC data indicating that Tp0624 is a functional monomer. Of the two molecules, chain A was the most completely modeled and was used for subsequent analyses unless otherwise stated. The overall architecture of Tp0624 is that of a thin triangle with approximate dimensions of 73 x 63 x 33 Å. Notably, the secondary structure elements are highly polarized with one side of the triangle comprised primarily of three beta-sheets and the opposite side dominated by ten alpha-helices (Fig 1B).

### Tp0624 adopts a highly modular architecture

**Fig 2:**
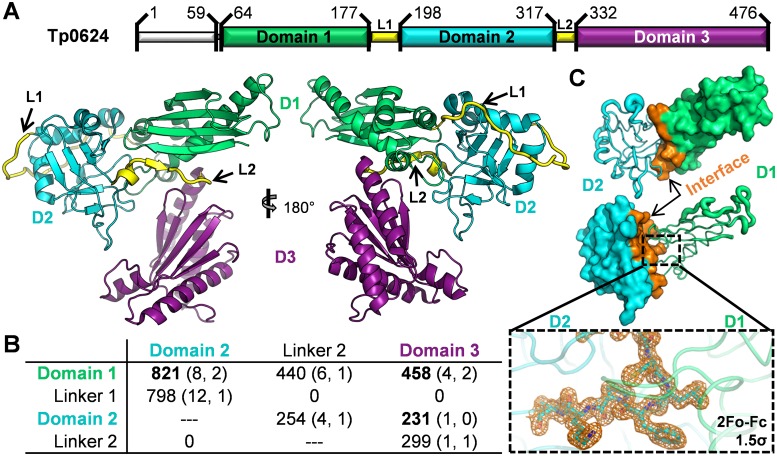
Tp0624 is organized into two units consisting of D1-D2 and D3. **(A)** Top: structure-based assignment of Tp0624 domains (D1, green; D2, cyan; D3, purple) and linkers (L1 and L2, yellow). Bottom: cartoon of Tp0624 tertiary structure colored by domain as in (top). **(B)** Table of inter-domain/linker interfaces of Tp0624 listed as “Interface Area in Å^2^ (# Hydrogen Bonds, # Salt Bridges)” as defined by PISA [37]. **(C)** Interface of D1-D2. Top: D2 (cyan) shown as B-factor defined coil with increased mobility indicated by thicker coil. D1 shown as green surface, with residues contacting D2 colored orange. Middle: Inverse display of (top). Inset: Sigma-A weighted electron density mesh (orange) contoured at 1.5 sigma for a loop of D2 (cyan) that protrudes into D1 (green).

Structural analysis reveals that Tp0624 is comprised of three distinct domains connected by two interdomain linkers: domain 1 (D1, A64-F177) is linked via a 20 residue linker (L1) to domain 2 (D2, Y198-C317), which is in turn linked via a 14 residue linker (L2) to domain 3 (D3, D332-D476) (Fig 2A). Linker 1 stretches across the back and around the end of D2 (Fig 2A), burying nearly 800 Å^2^ at the interface and forming 12 hydrogen bonds with D2 (Fig 2B) [37]. The N-terminal portion of L1 is well ordered while the C-terminal portion that wraps around the end of D2 displays significant flexibility. Despite the observed flexibility in L1, the buried interface and ordered residues between D1 and D2 suggest that these two domains function as an integrated unit (Fig 2B and 2C). While L2 connects D2 and D3, it also forms the terminal strand in the D1 beta-sheet highlighting the overall integrated nature of the Tp0624 modular architecture.

The C-terminal domain, D3, which is classified based on sequence to be part of the OmpA-OmpF porin (OOP) family [20], is the largest of the three domains and is formed by a five-stranded beta-sheet of mixed parallel and anti-parallel strands in the pattern 1-2-5-3-4 flanked on one face by three alpha-helices (Fig 2A). The core of the central domain, D2, adopts a pseudo beta-sandwich fold, with a top sheet of four parallel strands, and a bottom twisted sheet of four mixed strands; the two sheets are in parallel planes but orthogonally packed (Fig 2A). The N-terminal domain, D1, of Tp0624 is comprised primarily of a four-stranded anti-parallel beta-sheet (1-2-4-3 connectivity), supplemented by a short fifth strand derived from L2, with an extended helix that packs diagonally against the full length of one face of the beta-sheet. This domain core is supplemented by three short helices packed at the base of the domain, with the first interfacing with D3, the second bracing against the D1 primary helix, and the third forming a substantial portion of the interface with D2 (Fig 2A).

### Tp0624 D3 adopts a divergent OmpA-like domain that is unable to bind the diaminopimelic acid of conventional peptidoglycan

**Fig 3:**
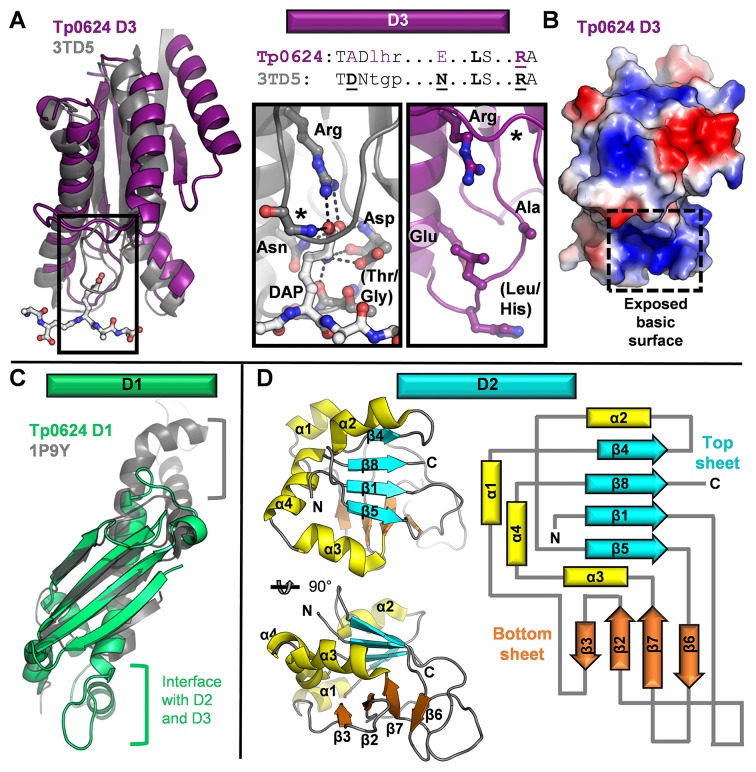
Comparative Tp0624 domain analyses. **(A)** Left: overlay of Tp0624 D3 (purple) on the OmpA-like domain of *Acinetobacter baumannii* (grey, PDB ID 3TD5) in complex with a peptidoglycan derivative (light grey ball-and-stick colored by element). Right: Structure-based sequence alignment of Tp0624 with the peptidoglycan binding residues of *A*. *baumanii* OmpA-like domain. Critical residues are bolded and displayed in the inset panels. Inset: Zoomed-in view of the peptidoglycan binding site on *A*. *baumanii* OmpA-like domain (grey, left), with hydrogen bonds to the diaminopimelic acid residue shown as dotted lines. Star indicates the loop that comprises the outer side of the pocket. Contrast with the same view of Tp0624 D3, which despite conservation of the essential Arg, shows the loss of a critical Asp to Ala, the displaced position of the outer loop (star), and the large residues (Leu/His) in the lower loop that project into the binding site. **(B)** Electrostatic surface of Tp0624 D3 in same orientation as (A) shows a clear basic patch comprising the putative ligand binding site. **(C)** Overlay of Tp0624 D1 (green) on the *E*. *coli* trigger factor (grey, PDB ID 1P9Y). Grey bracket, region of the trigger factor that has been functionalized for ribosome binding and is not conserved in Tp0624 D1. Green bracket, region of Tp0624 D1 that has been extended to form the interfaces with D2 and D3. **(D)** Tp0624 D2 is a previously uncharacterized domain showing no significant structural similarity to any domain in the PDB. Tp0624 consists of a four-stranded parallel top sheet (cyan), four-stranded mixed bottom sheet (orange), and flanking helices (yellow). Left: orthogonal views of Tp0624 D2 tertiary structure. Right: Topology diagram of Tp0624 D2.

To gain additional insight into potential functions of the three Tp0624 domains, we searched for related structures using the DALI server [38]. We first analyzed D3, which showed strong structural homology to the C-terminal domain of *E*. *coli* Outer Membrane Protein A (OmpA), which contributes to cell envelope stability by providing a structural link between the peptidoglycan layer and outer membrane [7–10, 12]. The closest structural homolog of Tp0624 D3 is the OmpA-like domain from *Vibrio* PomB, an inner membrane-anchored MotB ortholog and component of the flagellar motor that anchors the stator to the peptidoglycan layer (PDB ID 3WPW, Z-score 17.0 with scores < 2 being spurious, rmsd of 2.4 Å over 135 positions). The function of all OmpA-like domains that have been characterized to date is centered on their coordination of peptidoglycan via a pocket at the base of the domain [11]. A structural overlay on an OmpA-like domain with a bound peptidoglycan fragment (Fig 3A; *Acinetobacter baumannii* OmpA-like domain, PDB ID 3TD5) reveals key differences between Tp0624 D3 and canonical OmpA-like domains. For example, a loop that forms the outer border of the peptidoglycan binding site in other OmpA-like domains is displaced by nearly 15 Å in Tp0624 (Fig 3A, asterisk). In addition, while the base of the binding site tends to be lined by small residues (e.g. Thr-Gly in PDB ID 3TD5), a Leu-His pair presents a possible steric hindrance to binding in the analogous region of Tp0624 (Fig 3A, insets). Sequence and structural analysis shows that binding of peptidoglycan in the pocket of OmpA-like domains depends on the motif T**D**(x)_7_**N**xxLSxx**R**A, with Asp2, Asn10, and Arg17 being the most critical and highly conserved binding residues [11]. In contrast, Tp0624 D3 homologs from spirochetes present a conserved T**A**D(x)_6_**E/Q**xxLSxx**R**A motif (Fig 3A). Most notably, the Asp residue that coordinates the head group of the peptidoglycan-specific diaminopimelic acid (DAP) is mutated to Ala in Tp0624 D3 and this Ala is completely conserved in all fifteen of the full-length Tp0624 spirochetal homologs (S2 Fig). These structural observations are consistent with our inability to demonstrate binding between DAP and Tp0624 in either DAP soaked crystals or by isothermal titration calorimetry.

Intriguingly, biochemical analyses of spirochete peptidoglycan, including that found in *T*. *pallidum*, have shown that DAP is not a detectable constituent for most spirochetes, with a few noted exceptions such as *Leptospira* spp. [39, 40]. The unique peptidoglycan of treponemes and most spirochetes is comprised of muramic acid, glucosamine, L-ornithine (DAP replacement in most spirochetes), D-glutamic acid, D/L-alanine, and sometimes glycine [39, 41, 42]. Inspection of the electrostatic surface of the putative binding site on Tp0624 D3 reveals a strongly basic region, consistent with the conservation of the critical Arg, but loss of the Asp to Ala (Fig 3B). The presence of an exposed pocket at the base of the domain that has been modified primarily by the loss of an acidic charge (responsible for coordinating the amine of the DAP headgroup; Fig 3A, left inset), makes it tempting to speculate that Tp0624 D3 might indeed anchor to the thin periplasmic peptidoglycan layer of *T*. *pallidum*, but that its divergently functionalized OmpA-like domain coordinates D-glutamic acid.

### The N-terminus of Tp0624 reveals functionally repurposed (D1) and previously uncharacterized (D2) domain architectures

In contrast to D3, D1 and D2 do not show significant sequence identity to any proteins of known structure or function. Structural analysis of D1 (Fig 3C) using the DALI server showed moderate alignment with the ribosome binding domain of *E*. *coli* trigger factor (e.g. PDB ID 1P9Y); despite a sequence identity of only 4%, these domains overlay with an rmsd of 3.3 Å over 79 positions, corresponding to a Z-score of 5.4. While the central four-stranded beta-sheet and primary helix are well conserved, both ends are structurally divergent with apparent functional consequences. Specifically, the presence of helices at the base of Tp0624 D1 uniquely enable the domain to pack against D2 and D3, while the opposite end of D1 is truncated compared to the trigger factor where it is used to engage the ribosome [43]. These differences suggest that, while Tp0624 has retained a conserved core scaffold, it is repurposed for a yet to be determined function. Notably, differential functionalization of this scaffold in bacteria is common, with modified versions forming part of proteins ranging from a modular lytic transglycosylase thought to be important for the assembly and maturation of the bacterial cell wall (PDB ID 4CFO, Z-score 5.1) [44], to a plasmid relaxase domain important for conjugative plasmid transfer (PDB ID 3L6T, Z-score 4.7) [45].

In striking contrast to the more than 1200 structural similarity hits for Tp0624 D1, no structural analogues of D2 were identified. Thus, the Tp0624 D2 fold can be described as a previously uncharacterized domain. The defining feature of this domain is its orthogonally packed four-stranded beta-sheets flanked on three sides by four alpha-helices (Fig 3D). The orthogonal orientation clearly distinguishes Tp0624 D2 from the beta-sandwiches of well-characterized pathogen proteins such as the *Plasmodium* 6-Cys and *Toxoplasma* SRS domains [46–49]. This new domain is assembled with a notably circuitous topology as the connectivity for the top and bottom sheets is 4-8-1-5 and 3-2-7-6, respectively, with the central two beta-strands of the top sheet derived from the N- and C-terminal sequence. No obvious pockets or charged patches are present on the surface, making it difficult to predict a specific function for Tp0624 D2. However, a possible functional interdependence of D1 and D2 is indicated by assessing the mobility of D2, which is tightly organized through the core beta-sandwich as well as the regions at the interface with D1, primarily helix 1 and a portion of the extended loop between beta-strands 1 and 2. In contrast, helix 2 positioned atop the beta-sandwich and the regions distal to the D1 interface are notably more flexible (Fig 2C).

### The modular assembly of Tp0624 is positively correlated with treponeme pathogenicity

**Fig 4:**
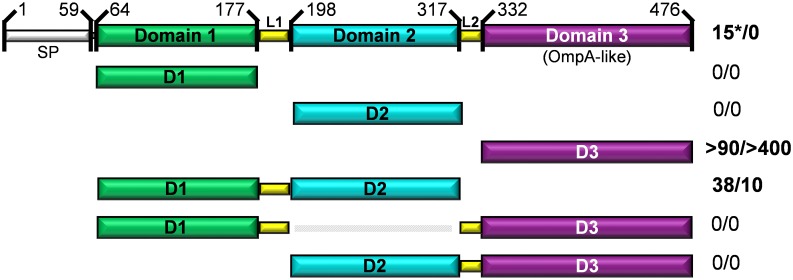
Tp0624 amino acid sequence-based modularity analysis. Orthologs of Tp0624 were identified using BLASTp. Tp0624 signal peptide (SP), domains (D1, green; D2, cyan; D3, purple), and linkers (L1 and L2, yellow) are shown. Indicated on the right are the numbers of orthologs with different domain combinations from spirochetes (left number) or other bacteria (right number). Sequences homologous to D1 and D2 only occur together, while D3 homologs are ubiquitous, and all three domains only occur together in treponemes (shown by an asterisk).

The intriguing modularity and domain architecture of Tp0624 indicate the possibility for functional interdependence, which led us to ask whether all three domains are reliant on each other for function or if specific domains are likely to act independently. To address these questions, we analyzed bacterial sequence databases using the domain boundaries established from the high-resolution structure (Fig 4). We found no examples of a sequence homologous to either D1 existing in isolation from D2 and D3, or D2 existing in isolation from D1 and D3. In contrast, sequences homologous to Tp0624 D3 were found in numerous proteins from both spirochetes and other bacteria that did not harbor domains homologous to D1 or D2. While the broad distribution of OmpA-like domains is not initially surprising, structural and binding studies reported here show clearly that Tp0624 D3 is unable to function as a canonical OmpA domain in binding conventional peptidoglycan.

The pattern of domain combinations is similarly intriguing as D1 and D2 were found together independently of D3 in nearly 50 protein sequences from a variety of bacteria consistent with the integrated nature of these domains in the crystal structure (Fig 2B and 2C) and further supporting that the D1-D2 pair is a functional unit. In contrast, the pairings of D2-D3 or D1-D3 were not observed in isolation (Fig 4). Perhaps most notably, the combination of all three domains together is only observed in treponemes, primarily in pathogenic species but also in three of the six non-pathogenic species (*T*. *primitia*, *T*. *azotonutricium*, and *T*. *caldaria*), suggesting that D3 can augment a treponeme-specific function of the D1-D2 pair. Next, we assessed the evolutionary relationship between the Tp0624 treponemal orthologs using full-length sequences or the three individual domains (S3–S6 Figs). Strikingly, an evolutionary separation emerged where the full-length Tp0624 appeared to be more closely related to orthologs from pathogenic treponemes relative to non-pathogenic treponemes (S3 Fig). However, when each of the three domains was analyzed individually, this clear phylogenetic division was lost (S4–S6 Figs) indicating that inclusion of all three Tp0624 domains in one protein positively correlates with treponemal pathogenicity.

### Putative mechanistic roles for Tp0624

**Fig 5:**
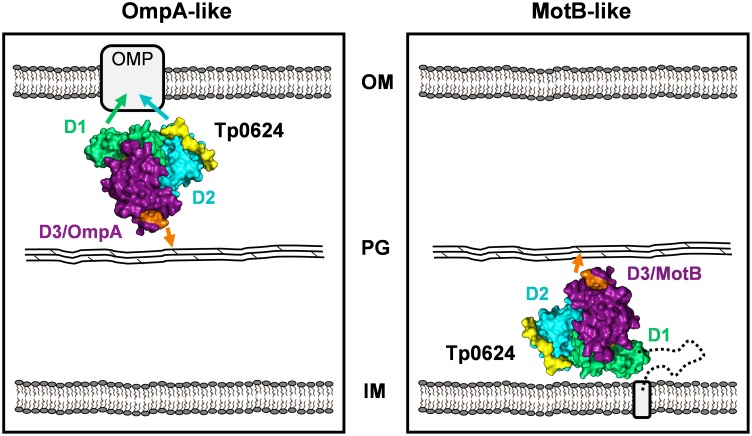
Model of the potential roles of Tp0624 in *T*. *pallidum* peptidoglycan binding and cell envelope structure stabilization. **(Left)** OmpA-like model: Tp0624 is exported across the inner membrane (IM) followed by cleavage and removal of the signal peptide. Tp0624 binds peptidoglycan (PG) via non-covalent interactions (orange arrow) involving the OmpA-like D3 while D1 and/or D2 interact with outer membrane-localized proteins (OMP). Residues from the treponemal conserved TAD(x)_6_E/QxxLSxxRA motif (putative non-canonical peptidoglycan binding motif) and surrounding pocket located within D3 are shown in orange **(Right)** MotB-like model: Tp0624 is translocated across the inner membrane, however the signal peptide is not cleaved and the protein remains anchored to the inner membrane, but with the functional head group directed into the periplasm. Tp0624 binds peptidoglycan via its OmpA-like domain, forming a structural bridge with the inner membrane. In this subcellular location, Tp0624 could function in cell envelope and/or flagellar stabilization.

Based on the data reported here, we propose two models describing potential subcellular locations and corresponding functional peptidoglycan binding roles for Tp0624 (Fig 5). In the first model, Tp0624 is exported across the inner membrane via the Sec-mediated protein translocation pathway. Following cleavage of the signal peptide, Tp0624 coordinates the thin peptidoglycan layer via the OmpA-like domain (D3) resulting in the D1/D2 tandem unit being positioned for interactions with outer membrane-localized proteins (Fig 5, left panel [OmpA-like]). In this model, Tp0624 performs a similar function to the *Neisseria meningitides* OmpA-like domain containing protein, RmpM (Reduction-modifiable protein M) [8, 9] to promote stabilization of the outer membrane. In the second model, Tp0624 is also exported from the cytoplasm, but is retained in the inner membrane through its non-cleavable, hydrophobic N-terminal signal peptide with its functional head group directed into the periplasm (Fig 5, right panel [MotB-like]). Similar to other flagellar motor complex proteins, such as MotB, Tp0624 would be positioned to form a structural bridge between the peptidoglycan layer and the inner membrane. Comparable to the class of OmpA domain-like-containing proteins such as MotB and PomB, Tp0624 may further help to stabilize the flagellar motor complex within the cell envelope by functioning as another immobilized structure against which the flagellar rotor and rod rotate. Since *T*. *pallidum* possesses a MotB homolog (Tp0724) within a flagellar operon [5] it is perhaps more likely that an inner membrane location for Tp0624 would allow the protein to function as a scaffolding structure to maintain correct peptidoglycan positioning within the cell envelope and this could be facilitated by the D1/D2 tandem unit [5]. Given that MotB is a stabilizing component of the flagellar motor complex, and is only located at the ends of the treponeme, other periplasm-directed, inner membrane-anchored proteins may be involved in maintaining correct peptidoglycan positioning and providing structural support along the length of the treponeme [5]; Tp0624 could be one example of a protein that functions in this manner.

## Conclusions

*T*. *pallidum* has a strategically minimalized complement of proteins, yet is one of the most invasive pathogens. Key virulence traits of *T*. *pallidum* include its unusual peptidoglycan structure, fragile cell envelope and internal endoflagella, yet the molecular strategies to functionalize and stabilize these ultrastructural features are largely unknown. In this study, we determined the high-resolution structure of Tp0624, and showed it to be comprised of a modular, three-domain architecture incorporating an OmpA-like domain and a tandem pair of previously uncharacterized domains. Intriguingly, the OmpA-like domain (D3) is sufficiently divergent that it is unable to bind the conventional DAP peptidoglycan component and may be uniquely adapted to coordinate the novel peptidoglycan structures of *T*. *pallidum* and potentially rigidify and stabilize the cell envelope. While we have not been able to establish a function for the previously uncharacterized domain pair (D1-D2), its association with D3 appears to correlate positively with treponeme pathogenicity and thus may play an important role in supporting the function of D3. Overall, our data suggests that Tp0624 may coordinate peptidoglycan through an unconventional mechanism to stabilize the inner- or outer-membranes of the highly divergent human pathogen, *T*. *pallidum*.

## Supporting Information

S1 Fig Size exclusion chromatogram of purified Tp0624. Superdex 75 HiLoad size exclusion chromatogram showing that Tp0624 (46 kDa) elutes as a monomer (elution peak of 44 kDa globular standard denoted by hash mark).
(PDF)

S2 Fig Amino acid alignment of full-length Tp0624 orthologs from treponemes. Orthologs of Tp0624 were identified using BLASTP (PSI-BLAST algorithm) and aligned in Clustal Omega and BioEdit sequence alignment editor. The predicted Tp0624 signal peptide cleavage site at T61-Q62 (SP), domains 1 (D1), 2 (D2), and 3 (D3), known OmpA domain peptidoglycan binding residues (TD(x)7NxxLSxxRA) and corresponding treponemal residues (red rectangles) are shown. Identical residues (black background), similar residues (gray background), and dissimilar residues (white background) are highlighted. At each position of the consensus sequence, an asterisk indicates full conservation among the 18 treponemes, a colon indicates strong conservation, and a dot indicates lower conservation.
(PDF)

S3 Fig Phylogenetic analysis of full-length treponemal Tp0624 orthologs. A phylogenetic tree of 18 full-length Tp0624 ortholog sequences from treponemes was inferred using the Neighbor-Joining method. The percentage of replicate trees in which the associated taxa clustered together in the bootstrap test (1000 replicates) was calculated. The tree was drawn to scale, with branch lengths in the same units as those of the evolutionary distances used to infer the phylogenetic tree. The evolutionary distances were computed using the JTT matrix-based method and were in the units of the number of amino acid substitutions per site. Evolutionary analyses were conducted using MEGA (Molecular Evolutionary Genetics Analysis) 6 software. The tree was rooted with the Tp0624 ortholog from *Spirochaeta thermophila* (Accession number: WP_013314620). The dashed line indicates the phylogenetic divide between pathogens (top) and non-pathogens (bottom).
(PDF)

S4 Fig Phylogenetic analysis of domain 1 from treponemal Tp0624 orthologs. A phylogenetic tree of Tp0624 ortholog sequences corresponding to domain 1 from 18 treponemes was inferred using the Neighbor-Joining method. The percentage of replicate trees in which the associated taxa clustered together in the bootstrap test (1000 replicates) was calculated. The tree was drawn to scale, with branch lengths in the same units as those of the evolutionary distances used to infer the phylogenetic tree. The evolutionary distances were computed using the JTT matrix-based method and were in the units of the number of amino acid substitutions per site. Evolutionary analyses were conducted using MEGA (Molecular Evolutionary Genetics Analysis) 6 software. The tree was rooted with the Tp0624 ortholog from *Spirochaeta thermophila* (Accession number: WP_013314620). Pathogens (P) and non-pathogens (NP) are indicated.
(PDF)

S5 Fig Phylogenetic analysis of domain 2 from treponemal Tp0624 orthologs. A phylogenetic tree of Tp0624 ortholog sequences corresponding to domain 2 from 18 treponemes was inferred using the Neighbor-Joining method. The percentage of replicate trees in which the associated taxa clustered together in the bootstrap test (1000 replicates) was calculated. The tree was drawn to scale, with branch lengths in the same units as those of the evolutionary distances used to infer the phylogenetic tree. The evolutionary distances were computed using the JTT matrix-based method and were in the units of the number of amino acid substitutions per site. Evolutionary analyses were conducted using MEGA (Molecular Evolutionary Genetics Analysis) 6 software. The tree was rooted with the Tp0624 ortholog from *Spirochaeta thermophila* (Accession number: WP_013314620). Pathogens (P) and non-pathogens (NP) are indicated.
(PDF)

S6 Fig Phylogenetic analysis of domain 3 from treponemal Tp0624 orthologs. A phylogenetic tree of Tp0624 ortholog sequences corresponding to domain 3 from 15 treponemes was inferred using the Neighbor-Joining method. The percentage of replicate trees in which the associated taxa clustered together in the bootstrap test (1000 replicates) was calculated. The tree was drawn to scale, with branch lengths in the same units as those of the evolutionary distances used to infer the phylogenetic tree. The evolutionary distances were computed using the JTT matrix-based method and were in the units of the number of amino acid substitutions per site. Evolutionary analyses were conducted using MEGA (Molecular Evolutionary Genetics Analysis) 6 software. The tree was rooted with the Tp0624 ortholog from *Spirochaeta alkalica* (Accession number: WP_018525408). Pathogens (P) and non-pathogens (NP) are indicated.
(PDF)
