# Supplementary material for: The Structure of Treponema pallidum Tp0624 Reveals a Modular Assembly of Divergently Functionalized and Previously Uncharacterized Domains
Source: PLoS One. 2016 Nov 10;11(11):e0166274. doi: 10.1371/journal.pone.0166274 (PMC5104382; doi:10.1371/journal.pone.0166274)
Supplement: S1 Fig — Superdex 75 HiLoad size exclusion chromatogram showing that Tp0624 (46 kDa) elutes as a monomer (elution peak of 44 kDa globular standard denoted by hash mark). (PDF) [file pone.0166274.s001.pdf]

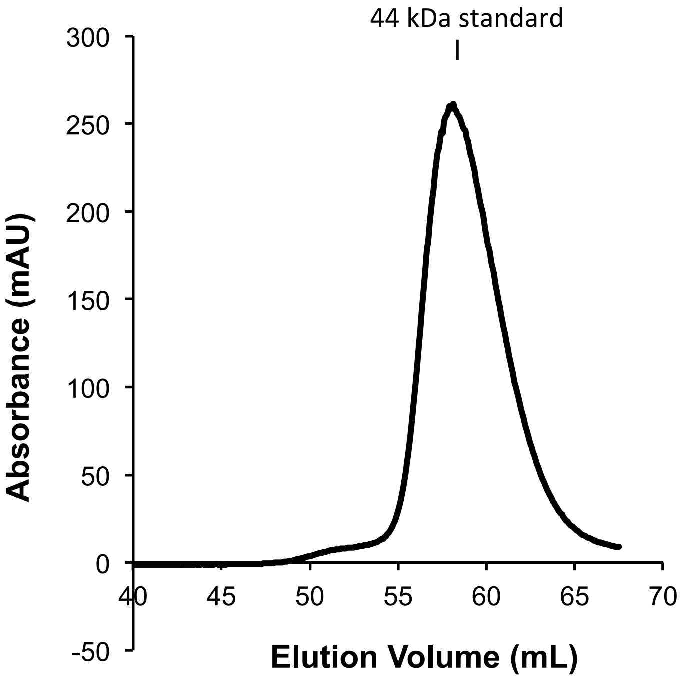

Supplementary Figure S6. Size exclusion chromatogram of purified Tp0624. Superdex 75 HiLoad size exclusion chromatogram showing that Tp0624 (46 kDa) elutes as a monomer (elution peak of 44 kDa; globular standard denoted by hash mark).
