# Supplementary material for: The Structure of Treponema pallidum Tp0624 Reveals a Modular Assembly of Divergently Functionalized and Previously Uncharacterized Domains
Source: PLoS One. 2016 Nov 10;11(11):e0166274. doi: 10.1371/journal.pone.0166274 (PMC5104382; doi:10.1371/journal.pone.0166274)
Supplement: S2 Fig — Orthologs of Tp0624 were identified using BLASTP (PSI-BLAST algorithm) and aligned in Clustal Omega and BioEdit sequence alignment editor. The predicted Tp0624 signal peptide cleavage site at T61-Q62 (SP), domains 1 (D1), 2 (D2), and 3 (D3), known OmpA domain peptidoglycan binding residues (TD(x)7NxxLSxxRA) and corresponding treponemal residues (red rectangles) are shown. Identical residues (black background), similar residues (gray background), and dissimilar residues (white background) are highlighted. At each position of the consensus sequence, an asterisk indicates full conservation among the 18 treponemes, a colon indicates strong conservation, and a dot indicates lower conservation. (PDF) [file pone.0166274.s002.pdf]

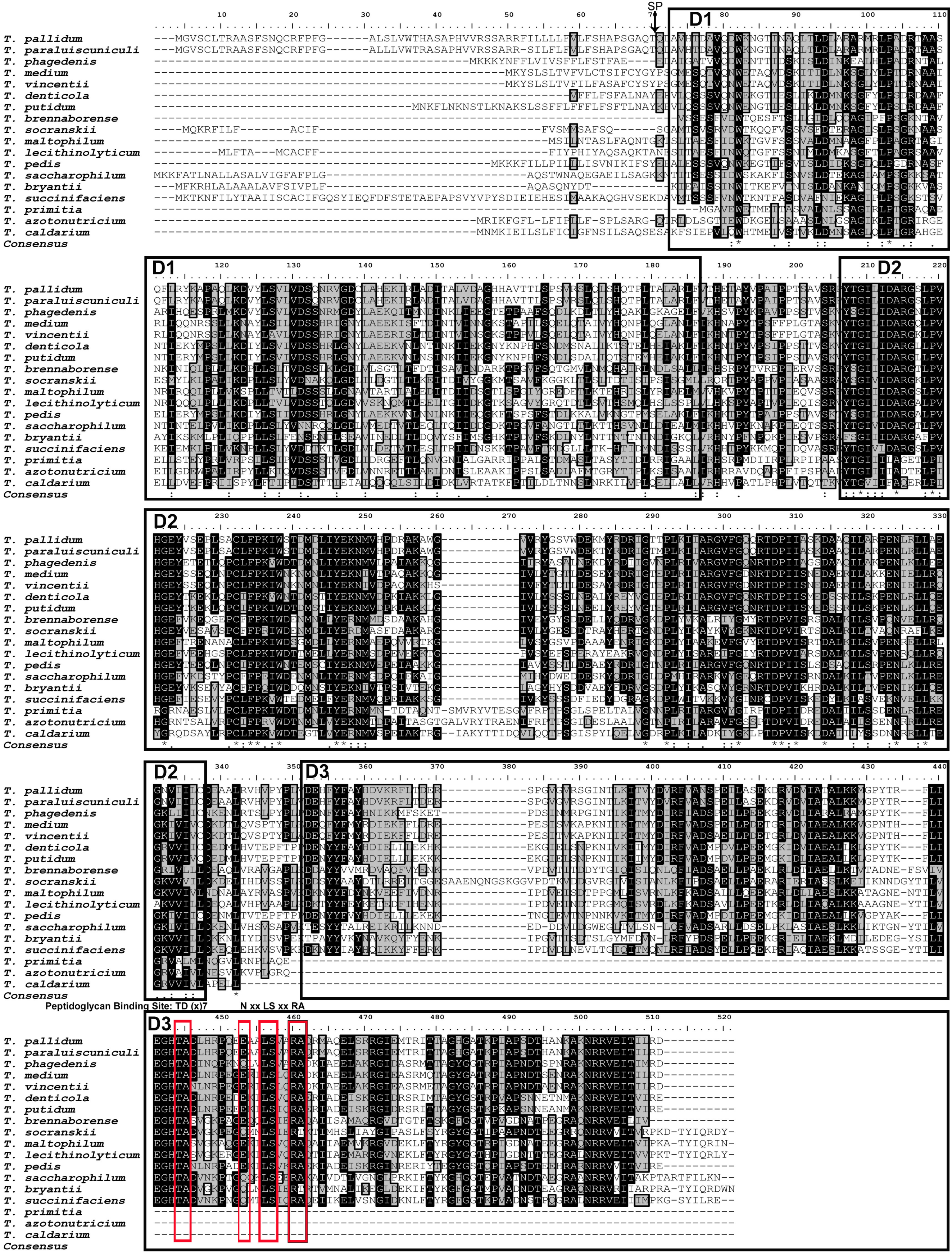

Supplementary Figure S2. Amino acid alignment of full-length Tp0624 orthologs from treponemes. Orthologs of Tp0624 were identified using BLASTP (PSI-BLAST algorithm) and aligned in Clustal Omega and BioEdit sequence alignment editor. The predicted Tp0624 signal peptide cleavage site at T61-Q62 (SP), domains 1 (D1), 2 (D2), and 3 (D3), known OmpA domain peptidoglycan binding residues (TD(x)7NxxLSxxRA) and corresponding treponemal residues (red rectangles) are shown. Identical residues (black background), similar residues (gray background), and dissimilar residues (white background) are highlighted. At each position of the consensus sequence, an asterisk indicates full conservation among the 18 treponemes, a colon indicates strong conservation, and a dot indicates lower conservation.
