# Supplementary material for: The Structure of Treponema pallidum Tp0624 Reveals a Modular Assembly of Divergently Functionalized and Previously Uncharacterized Domains
Source: PLoS One. 2016 Nov 10;11(11):e0166274. doi: 10.1371/journal.pone.0166274 (PMC5104382; doi:10.1371/journal.pone.0166274)
Supplement: S3 Fig — A phylogenetic tree of 18 full-length Tp0624 ortholog sequences from treponemes was inferred using the Neighbor-Joining method. The percentage of replicate trees in which the associated taxa clustered together in the bootstrap test (1000 replicates) was calculated. The tree was drawn to scale, with branch lengths in the same units as those of the evolutionary distances used to infer the phylogenetic tree. The evolutionary distances were computed using the JTT matrix-based method and were in the units of the number of amino acid substitutions per site. Evolutionary analyses were conducted using MEGA (Molecular Evolutionary Genetics Analysis) 6 software. The tree was rooted with the Tp0624 ortholog from Spirochaeta thermophila (Accession number: WP_013314620). The dashed line indicates the phylogenetic divide between pathogens (top) and non-pathogens (bottom). (PDF) [file pone.0166274.s003.pdf]

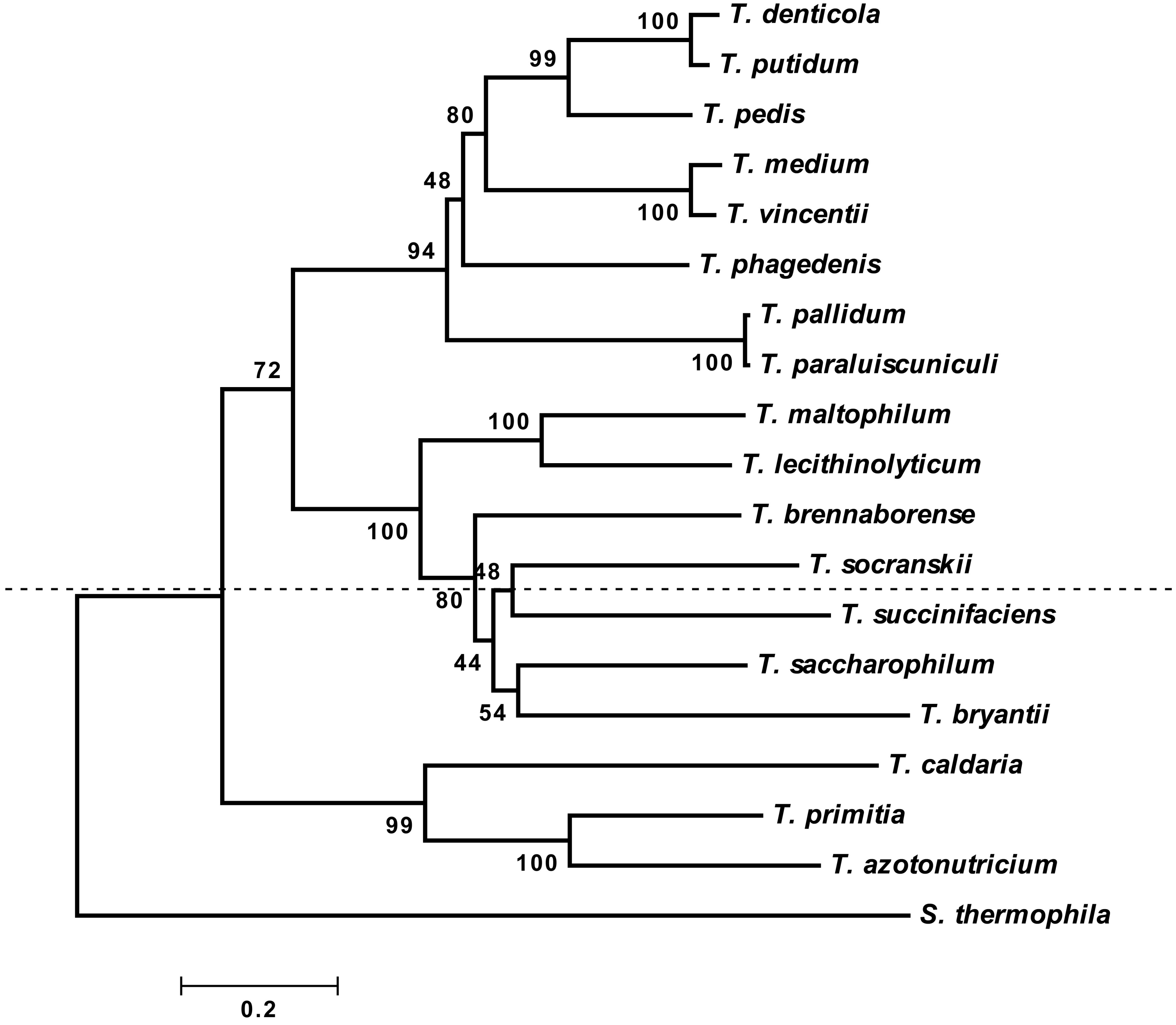

Supplementary Figure S3. Phylogenetic analysis of full-length treponemal Tp0624 orthologs.

A phylogenetic tree of 18 full-length Tp0624 ortholog sequences from treponemes was inferred using the Neighbor-Joining method. The percentage of replicate trees in which the associated taxa clustered together in the bootstrap test (1000 replicates) was calculated. The tree was drawn to scale, with branch lengths in the same units as those of the evolutionary distances used to infer the phylogenetic tree. The evolutionary distances were computed using the JTT matrix-based method and were in the units of the number of amino acid substitutions per site. Evolutionary analyses were conducted using MEGA (Molecular Evolutionary Genetics Analysis) 6 software. The tree was rooted with the Tp0624 ortholog from *Spirochaeta thermophila* (Accession number: WP_013314620). The dashed line indicates the phylogenetic divide between pathogens (top) and non-pathogens (bottom).
