# Supplementary material for: The Structure of Treponema pallidum Tp0624 Reveals a Modular Assembly of Divergently Functionalized and Previously Uncharacterized Domains
Source: PLoS One. 2016 Nov 10;11(11):e0166274. doi: 10.1371/journal.pone.0166274 (PMC5104382; doi:10.1371/journal.pone.0166274)
Supplement: S5 Fig — A phylogenetic tree of Tp0624 ortholog sequences corresponding to domain 2 from 18 treponemes was inferred using the Neighbor-Joining method. The percentage of replicate trees in which the associated taxa clustered together in the bootstrap test (1000 replicates) was calculated. The tree was drawn to scale, with branch lengths in the same units as those of the evolutionary distances used to infer the phylogenetic tree. The evolutionary distances were computed using the JTT matrix-based method and were in the units of the number of amino acid substitutions per site. Evolutionary analyses were conducted using MEGA (Molecular Evolutionary Genetics Analysis) 6 software. The tree was rooted with the Tp0624 ortholog from Spirochaeta thermophila (Accession number: WP_013314620). Pathogens (P) and non-pathogens (NP) are indicated. (PDF) [file pone.0166274.s005.pdf]

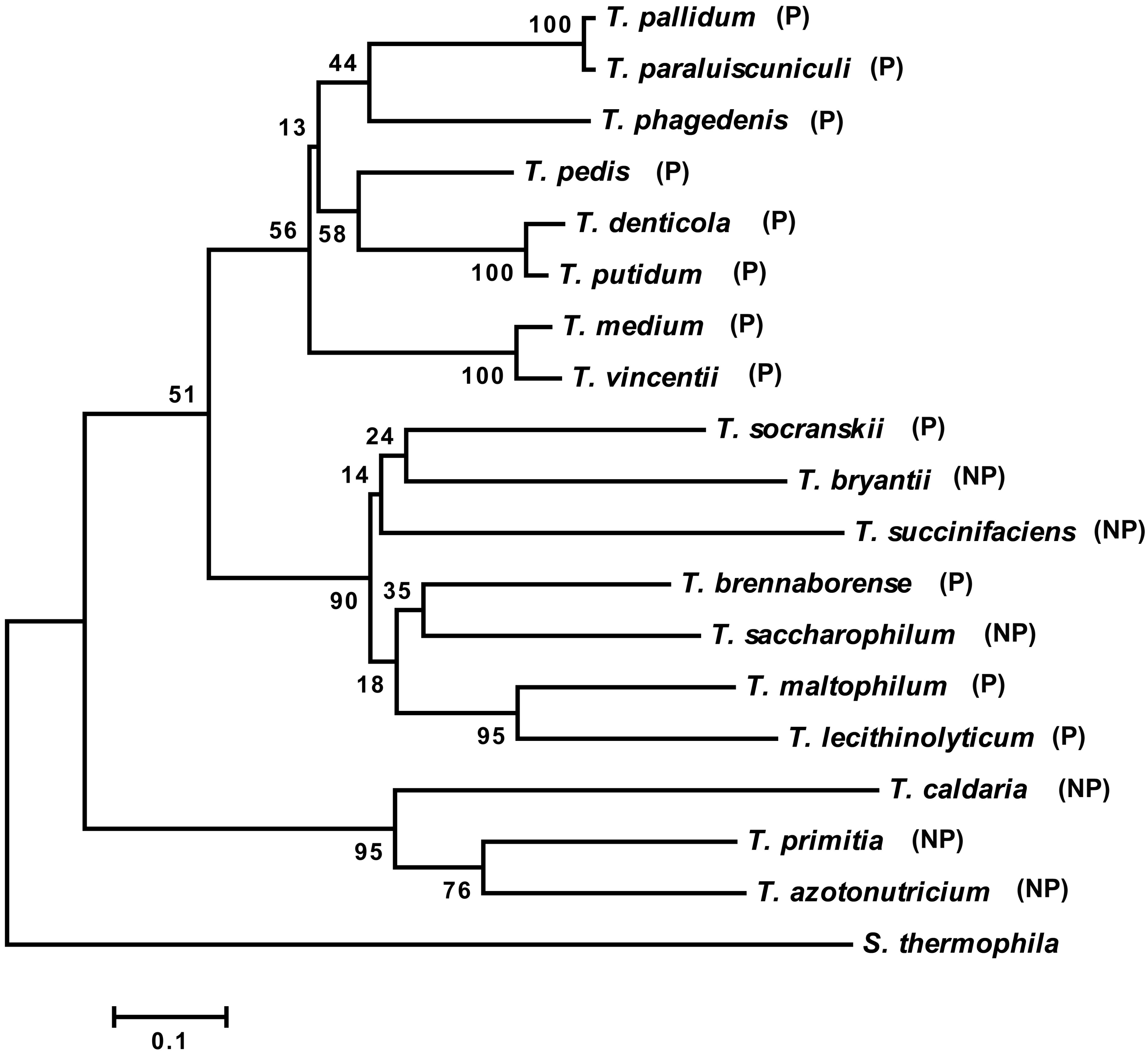

Supplementary Figure S5. Phylogenetic analysis of domain 2 from treponemal Tp0624 orthologs.

A phylogenetic tree of Tp0624 ortholog sequences corresponding to domain 2 from 18 treponemes was inferred using the Neighbor-Joining method. The percentage of replicate trees in which the associated taxa clustered together in the bootstrap test (1000 replicates) was calculated. The tree was drawn to scale, with branch lengths in the same units as those of the evolutionary distances used to infer the phylogenetic tree. The evolutionary distances were computed using the JTT matrix-based method and were in the units of the number of amino acid substitutions per site. Evolutionary analyses were conducted using MEGA (Molecular Evolutionary Genetics Analysis) 6 software. The tree was rooted with the Tp0624 ortholog from *Spirochaeta thermophila* (Accession number: WP_013314620). Pathogens (P) and non-pathogens (NP) are indicated.
